# Active consideration in an emotional context: implications for information processing

**DOI:** 10.3389/fpsyg.2024.1367714

**Published:** 2024-06-20

**Authors:** Sophie Jakob, Kai Hamburger

**Affiliations:** Experimental Psychology and Cognitive Science, Department of Psychology, Justus Liebig University, Gießen, Germany

**Keywords:** accuracy prompts, *Inaccuracy Prompts*, news framing, disinformation, emotional cues, fake news, consciousness, dual process theory

## 1 Introduction

In contemporary cognitive science, the interplay between emotions and information processing is crucial, especially in the context of media and misinformation. Previous research has extensively explored socio-affective factors influencing information processing and suggests that our cognitive and emotional capacities are not solely internal but are scaffolded by dynamic interactions with our material and social environments (Sterelny, 2010; Colombetti and Krueger, 2015). A particular emphasis in recent research focuses on cognitive drivers for the acceptance of false information (Ecker et al., 2022). Dual-process theories, such as motivated cognition (Kahan, 2017) and classical reasoning, provide foundational frameworks for understanding the role of analytical thinking in shaping beliefs (Pennycook and Rand, 2019). Additionally, the influence of emotions and framing of news on information processing has been a focal point, with studies showcasing the significant impact of emotional language on belief formation and sharing of news (Martel et al., 2020; Roozenbeek et al., 2022). Building upon the existing literature, this opinion article expands on the role of emotional cues and their influence on cognitive mechanisms. Within this framework, this paper introduces the concept of “Inaccuracy Prompts”. These prompts, akin to their accuracy counterparts, are posited to sway individuals toward less critical thinking, thus contributing to the acceptance of misinformation.

## 2 The interplay of emotions and rational decision-making systems

Rational decision-making, traditionally seen as separate from emotion and deeply rooted in Western philosophy, as advocated by figures like Aristotle, who advocated for a strict separation between emotion and rational action. Decision-making involves weighing alternatives, beliefs about outcomes, and values, yet conventional research often simplifies the subjective, emotional experiences of decision-makers, ignoring the complexity inherent in real-world decisions (Strle, 2016). Modern cognitive research refutes the strict separation of emotion and rationality, demonstrating that emotions are essential to rational processes and that no decision can occur without emotional involvement (Damásio, 2001; Bonansinga, 2020). Empirical evidence suggests that effective decision-making is not merely a product of logical deliberation but also relies on the intricate interplay of emotional insights, as emotions emerge from the complex interplay of more basic psychological ingredients such as core affect and conceptualization, which integrate bodily sensations and context-dependent interpretations to form discrete emotions (Lindquist, 2013). Kahneman (2011) was one of the most popular proponents of this theory. He divided our cognitive processes into two systems with regards to how we process and evaluate information and subsequently make our decisions: System 1, which is fast, intuitive, emotion-based, and unconscious, and System 2, which is slow, deliberate, controlled, and conscious (i.e., rational). Dual-process theories tend to view emotion and intuition on the one hand, and logic and reason on the other, as dichotomous opposites. This view is now controversial and is challenged by the enactive approach which emphasizes that perception and action are deeply integrated, where the mind is not merely reacting to but is actively shaped by its interactions within an environment (Colombetti, 2007). The *Affect as Information Theory* (AIT), as well assumes an interdependent connection between cognition and emotion (Clore et al., 2001; Clore and Huntsinger, 2007). Gigerenzer (2008) further enriches this perspective by illustrating that simple heuristics, often stemming from intuitive processes, can outperform complex cognitive operations, particularly under uncertainty. *Affective Intelligence* postulates that emotions, particularly feelings of (in)security, give us feedback about unconscious processes and play a role in both intuitive and deliberative judgment (Marcus et al., 2019). Furthermore, AIT posits that increased anxiety favors explicit reasoning in uncertain situations, while the absence of anxiety suggests reliance on habitual decisions in familiar contexts (MacKuen et al., 2007; Marcus et al., 2019). The concept of the *scaffolded mind* posits as well that our mental processes are extensively supported not just internally but through our continuous interaction with the physical and social environment (Sterelny, 2010; Colombetti and Krueger, 2015). It highlights how emotions and rational systems are interwoven and the significant role our surroundings play in shaping cognitive and emotional outputs.

In short, human judgments can easily be influenced by emotional factors, even if they are not necessarily related to the current situation.

## 3 Emotional language and framing in news media and political discourse

The following section examines the pivotal role of emotional language and framing within news media and political discourse, and how these elements influence public perception and the spread of political misinformation. Emotions are not just reactionary; they are instrumental in shaping our judgments and perceptions, particularly in the context of political information that often employs emotional triggers to influence and mislead (Martel et al., 2020).

Rather than consciously querying their feelings, individuals inherently integrate affectivity within the context of decision-making, as it naturally influences the evaluation of information (Damásio, 2001; Schwarz, 2012). Affective feelings, both positive and negative, have a variable influence on cognitive processing styles available (e.g., heuristic versus systematic; Huntsinger and Ray, 2016). Drawing on the concept of the *scaffolded mind* (Colombetti and Krueger, 2015), the role of environmental scaffolding can be applied to understand how news media utilize emotional language to create *affective niches* that manipulate public perception. These niches, strategically amplify specific emotional responses that can facilitate the spread of misinformation. This scaffolding of the affective mind by media channels serves not only to inform but to evoke and sustain particular emotional states that can skew rational decision-making processes. Considering Roozenbeek et al. (2022), emotional language significantly increases the likelihood of misinformation being shared and believed, demonstrating why such language is identified as a key manipulation technique in the spread of false information. Compared to neutral content, emotionally charged information attracts our attention more strongly and can have a distorting effect on our perception (Zajonc, 1984; Schwarz, 2012; Ecker et al., 2022). A popular example are short-form video platforms like TikTok. It attracts our attention more strongly and immediately exposes viewers to provocative content that enhances emotional responses and engagement (Cheng and Li, 2024). Anger in particular spreads faster than any other emotion on social media platforms (SMP) and contributes to the virality of fake news (Fan et al., 2014; Corbu et al., 2021; Michel and Gandon, 2024) and due to the negativity bias, negative information leave a stronger memory trace than positive information (Courbet et al., 2014). In combination with recommendation algorithms on SMPs, negative emotions provide fertile ground for the spread of false information in particular (Roozenbeek et al., 2022; Michel and Gandon, 2024).

In the political realm, disinformation campaigns use emotionally charged language to capture attention, reinforce messages, and provoke specific reactions, aiming to deepen emotional engagement and influence perceptions and responses to content more intensely (Corbu et al., 2021). One tool to achieve this is to frame information differently. Frames can roughly be divided into two categories: thematic and episodic (Gross, 2008; Aarøe, 2011). Thematic frames focus on presenting political issues within a broader context, offering abstract and general evidence. In contrast, episodic frames illuminate issues through concrete events and particular cases, providing specific characters at which emotional reactions can be directed (Gross, 2008; Aarøe, 2011). Episodic frames not only trigger a stronger emotional response. These emotions are also suitable for directing the impact of this emotion into support for an argued policy position (Aarøe, 2011).

Research shows the effectiveness of this kind of emotional manipulation in spreading misinformation. For instance, Peters et al. (2009) found that participants were more willing to share social anecdotes that aroused interest, surprise, disgust, and happiness with an unspecified audience. Conversely, those with low emotional reactivity were more inclined to overlook or distance themselves from the propagation of false information (Horner et al., 2021). These effects are notably significant for politically charged topics traditionally associated with emotive media language, often sparking public debates and controversies.

## 4 Call for research on the risk of active consideration as an *Inaccuracy Prompt*

Influential information sources like news media and politicians play a crucial role in shaping public perception. Politicians often demand of each other to “be aware” of certain things in their debates, meaning to “be attentive,” “to engage,” and to “actively consider.” Therefore, we will refer to *active consideration* as a form of attentiveness, engagement, and critical evaluation. The call to focus our awareness and attention on certain situations and information seems to make sense, since current political disinformation campaigns are an increasing concern (Lewandowsky et al., 2020). In fact, prompting people to stop and think about an issue at hand is a popular approach in current science with the aim of counteracting the spread of misinformation.

One way to achieve this is using so-called *Accuracy Prompts* (APs; Pennycook and Rand, 2021). APs are a nudge intervention technique aimed at making individuals aware of the concept of accuracy in news headlines, thereby boosting their ability to differentiate true from false information and reducing their susceptibility to believing and sharing false information (Pennycook et al., 2023). APs are therefore cues for users to enhance their critical thinking (e.g., assess the accuracy of information Pennycook and Rand, 2022; Capraro and Celadin, 2023) and intend to cause a reader to shift from superficial, intuitive System 1 thinking to critically reflective System 2 thinking (Evans and Stanovich, 2013; Pennycook and Rand, 2019). Or, to put it another way, APs encourage people to be more aware about an issue at hand. Although the general effectiveness of APs has been widely documented (Bhardwaj et al., 2023; Pennycook et al., 2023), we found that there is no explicit research on whether and how the impact of APs is influenced by the framing of messages and the associated emotional context.

In general, APs aim to guide people from System 1 toward deliberate System 2 thinking. However, we theorize that expressions like “we need to be aware of the fact that...” might reverse this effect and instead draw the attention of individuals to the emotional backdrop of presented information, thereby turning an AP into something we coin as *Inaccuracy Prompt* (IAP). By our definition, IAPs share the same goal as APs but fail to guide the reader's attention to the concept of accuracy and instead boost their awareness of their own affective response to news, influenced by the emotional context and framing in which the information is presented. In contrast to APs, IAPs are not always designed as explicit interventions and can occur by accident. In summary, prompts aimed to enhance accuracy could function as both Accuracy- and Inaccuracy cues, depending on framing and elicited emotions. Specifically, when APs explicitly call for active consideration in an emotionally charged, episodically framed context, they may amplify emotional salience, potentially promoting the spread of disinformation. Emotions, especially anger and enthusiasm, mediate framing effects and foster reliance on heuristics (Bodenhausen et al., 1994; Marcus et al., 2000; Lecheler et al., 2013). Anger in particular promotes the tendency to believe politically motivated misinformation (Weeks, 2015).

**Figure 1:**
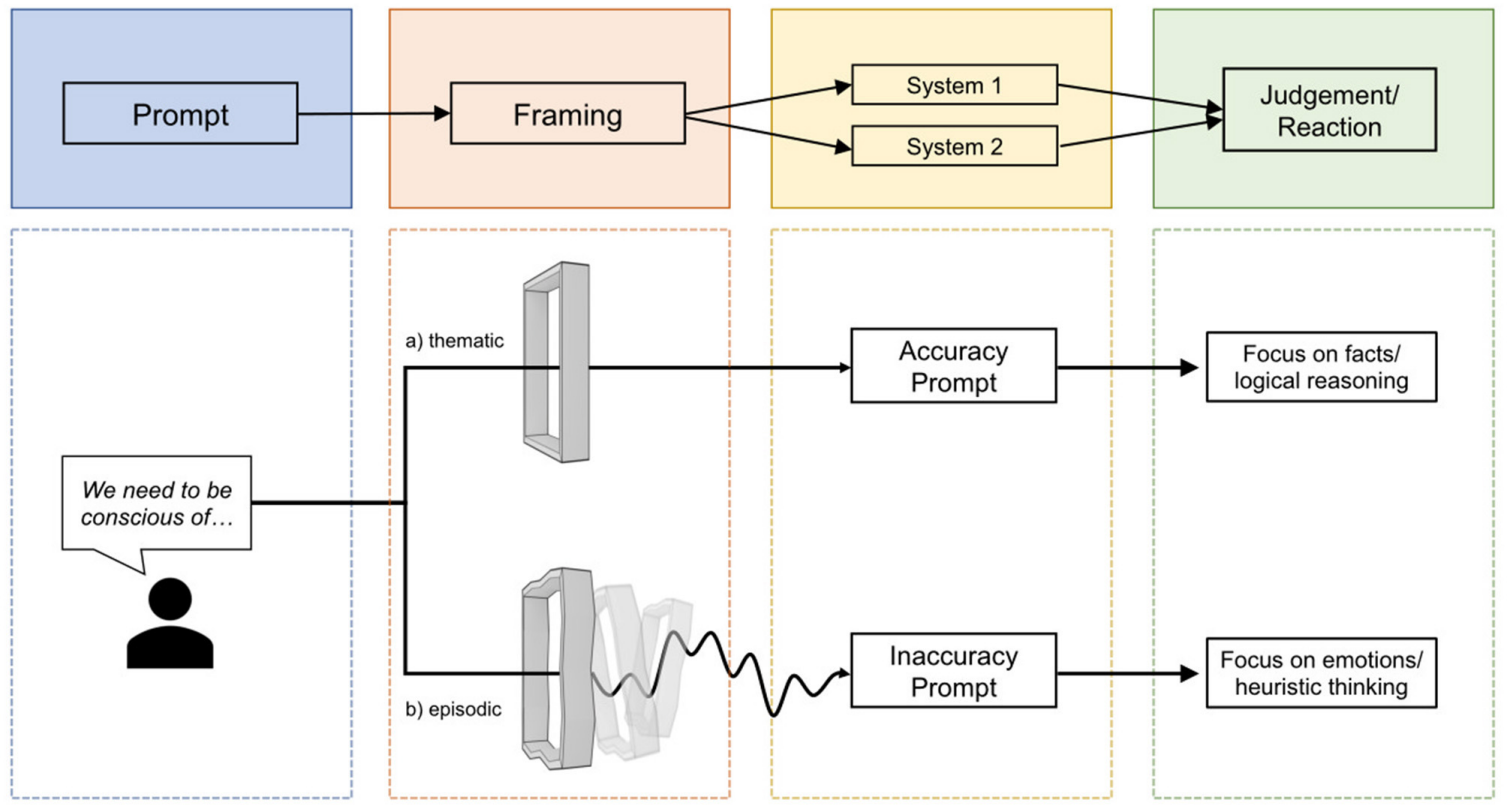
Whether the evaluation or response to information relies more on facts or emotions depends on the framing in which the relevant information is embedded.

The challenge is that while people are encouraged to think critically (what is being said), the AP might draw attention to the emotionally loaded context in which these statements are embedded – reflecting how our emotional and cognitive capacities are not merely internal but are shaped by our interactions with both material culture and social relationships; i.e., *how* it is being said (Figure 1).

We posit that this might cause two possible effects: (1) masking the strength of emotional influences or, if you consider the activating properties of anger (which populists, for example, like to exploit) (2) act as some sort of magnifying glass, causing people to double down on their opinions. In any case, these concerns motivate our call for research on possible interactions between APs, framing and discrete emotions.^1^

Another reason for our assumption is that urging individuals to engage in more active or considered thought may paradoxically result in lower-quality decision-making (Dijksterhuis and Nordgren, 2006). Researchers recognize the challenges in decision-making due to the limited capacity of conscious processing, which can lead to suboptimal choices (Tversky and Kahneman, 1974; Kahneman, 2003). Considered thought, intricately linked to attention and the resulting decisions, confronts constraints imposed by the limited capacity of verbal working memory (Baddeley, 1992), which can only temporarily “store” approximately four information units (Wilhelm et al., 2013). Particularly considering the increasing “infodemic”, facilitated by the internet (Corbu et al., 2021; Bortolotti, 2023), considered thought consequently can only focus on a subset of the information that should be accounted for, potentially leading to suboptimal decisions (Simon, 1955; Tversky and Kahneman, 1974; Kahneman, 2003). This assumption is supported by experimental studies that investigated the quality of the decisions people made when they had to make either conscious or unconscious decisions about complex issues. Compared with people who thought less, people who engaged in this *consciousness by necessity* (Dijksterhuis and Nordgren, 2006) made less accurate evaluations, suggesting that conscious (i.e. active or considered) thought led them to focus on a limited number of attributes at the expense of taking into account other relevant attributes.

The possible negative influence of conscious thought on decisions does not necessarily contradict the AP-approach. Rather, this data illustrates the importance to focus our limited awareness capacities on important core aspects (i.e., facts) of controversial issues in contrast to the emotional load they might be embedded in. In summary, both the influence of emotions and framing effects on news perception and the dissemination of misinformation, as well as APs as an intervention against the latter, are subject of current research. However, in our view, there is a data gap regarding the question of whether and how an emotional context could potentially influence, negate, or even inverse the effect of accuracy prompts (i.e., IAP); a gap we would like to address in future research.

## 5 Quo Vadis?

Examining emotional language and framing in news media and political discourse highlights the powerful role emotions play in shaping perceptions, especially in the context of political fake news and disinformation campaigns, challenging the traditional dichotomy between emotions and rationality. Considering the rampant misinformation and fake news prevalent in today's information landscape, understanding the intricate interactions between accuracy prompts, framing, and emotions becomes paramount. Negative emotions like anger not only attract attention but also reinforce cognitive biases, contributing to the spread of fake news (Martel et al., 2020; Corbu et al., 2021). The construction of filter bubbles by recommendation systems further amplifies this effect, trapping users into echo chambers of emotional states (Fan et al., 2014; Corbu et al., 2021; Chuai et al., 2023; Michel and Gandon, 2024). While APs aim to direct attention toward accuracy, the influence of emotions on engagement and credibility assessment remains a critical research gap (Martel et al., 2020). Understanding these dynamics is essential for addressing the spread of misinformation and promoting critical thinking.

Building on previous research, we suggest investigating the notion of IAPs. Specifically, we theorize that calling for active considerations, when immersed in an emotionally charged environment, might unintentionally steer individuals toward less critical thinking, potentially fostering the acceptance of misinformation.

The discussion on the limited capacity of information processing, with working memory being the “bottle neck,” highlights the need to focus on essential aspects of issues within emotional contexts (Tversky and Kahneman, 1974; Baddeley, 1992; Kahneman, 2003; Wilhelm et al., 2013). Complementing this, Oblak et al. (2024) suggest that understanding background feelings and transmodal dynamics during working memory tasks can elucidate variations in performance and overall conscious experience. Investigating the dynamics of how emotional cues, consciousness, and information prompts interact may provide essential insights into the dynamics of information dissemination and user engagement on SMPs and thereby shaping public perception and decision-making.

The potential dual function of prompts, contingent upon contextual factors, introduces complexity to information dissemination strategies. Ultimately, this exploration could contribute to the ongoing discourse on promoting critical thinking and improving decision-making quality within emotionally charged information environments.

## Author contributions

SJ: Visualization, Writing – original draft, Writing – review & editing. KH: Supervision, Writing – review & editing.

## Footnotes

^1^In the context of this paper we define distinct emotions as the basic emotions described by Ekman and Cordaro (2011) which encompass Happiness, Sadness, Fear, Anger, Disgust, Contempt, and Surprise.
